# Epidemiological Survey of Different Treatments for Choledocholithiasis in Taiwan: A Nationwide, Population-Based Cohort Analysis

**DOI:** 10.3390/jcm11040970

**Published:** 2022-02-12

**Authors:** Jia-Hui Chen, Chi-Hsiang Chung, Chung-Hsien Li, Wu-Chien Chien, Chao-Feng Chang

**Affiliations:** 1 Division of General Surgery, Department of Surgery, Taipei Tzu Chi Hospital, The Buddhist Medical Foundation, New Taipei City 231, Taiwan; procto77@gmail.com; 2 School of Medicine, Buddhist Tzu Chi University, Hualien 970, Taiwan; 3 School of Public Health, National Defense Medical Center, Taipei City 114, Taiwan; g694810042@gmail.com; 4 Taiwanese Injury Prevention and Safety Promotion Association, Taipei City 114, Taiwan; 5 Division of Gastroenterology, Department of Medicine, Taipei Tzu Chi Hospital, The Buddhist Medical Foundation, New Taipei City 231, Taiwan; top920819@gmail.com; 6 Department of Medical Research, Tri-Service General Hospital, National Defense Medical Center, Taipei City 114, Taiwan; 7 Graduate Institute of Life Sciences, National Defense Medical Center, Taipei City 114, Taiwan; 8 Division of Gastroenterology, Department of Internal Medicine, Tri-Service General Hospital, National Defense Medical Center, Taipei City 114, Taiwan

**Keywords:** choledocholithiasis, endoscopic sphincterectomy, laparoscopic choledocholithtomy, population-based study, Taiwan

## Abstract

(1) Background: Open, laparoscopic, and endoscopic choledocholithotomy (OC, LC, and EC, respectively) are accepted choledocholithiasis treatment modalities. However, an assessment of the nationwide trends in their outcomes is lacking. This nationwide population-based analysis evaluated treatment outcomes of choledocholithiasis in Taiwan; (2) Methods: A total of 13,139,306 individuals were randomly enrolled from the Longitudinal Health Insurance Database (LHID) between 2000 to 2013 for cohort analysis. All patients with newly diagnosed choledocholithiasis aged 18 years or older who were treated during the study period were enrolled and allocated to the OC, LC, EC, or combined endoscopy and open choledocholithotomy (CEOC) groups. Age, readmission, retained stone, comorbidities, hospital stay, medical cost, complications, mortality were analyzed; (3) Results: A total of 58,064 individuals met the inclusion criteria, including 46.54%, 1.10%, 47.52%, and 4.85% who underwent OC, LC, EC, and CEOC, respectively. The endpoint characteristics showed that the LC group had higher readmission, longer hospital stay, and higher medical cost. Cox regression analysis showed that the adjusted hazard ratio (HR) of complications for EC was 1.259 times higher than that for OC. The adjusted HRs of readmission within 90 days for LC, EC, and CEOC were higher than that of OC. The adjusted HR of retreatment with surgery was higher in LC. The adjusted HR of retreatment with endoscopy was higher in CEOC. The adjusted HR of mortality in EC was 1.603 times that of OC; (4) Conclusions: Different choledocholithiasis treatments lead to different outcomes. However, further studies on other large or national data sets are required to support these findings.

## 1. Introduction

Cholelithiasis occurs in 10–15% of the general population [1]. Between 10 and 18% of patients undergoing laparoscopic cholecystectomy for cholelithiasis have synchronous choledocholithiasis [2]. No unanimous consensus has been achieved regarding the ideal management of choledocholithiasis. The old and new treatment approaches include open surgery, laparoscopy, and endoscopy [2,3,4].

The goal of treatment in choledocholithiasis is to achieve ductal clearance with the fewest number of interventions, least morbidity, and lowest costs. Previous evidence suggests that open choledocholithotomy (OC) is better than endoscopic choledocholithotomy (EC) in achieving common bile duct stone clearance based on evidence during the early endoscopy period [5]. The mortality and morbidity between laparoscopic choledocholithotomy (LC) and EC options show no significant difference [5,6]. In comparison with LC, pre-operative and intra-operative EC has no significant reduction in the number of retained stones and failure rates [5,7]. There is also no significant difference in the failure rates, retained stones, morbidity, and mortality between single-stage LC and two-stage EC treatment [5,6,7,8,9].

However, estimates of the population-based outcomes of different choledocholithiasis treatments and their related factors are lacking. Therefore, we analyzed the Taiwan National Health Insurance Research Database to assess the 14-year trend in efficiency, safety, and outcomes of surgical and endoscopic treatment of choledocholithiasis.

## 2. Materials and Methods

This study analyzed data from the Taiwan Longitudinal Health Insurance Database (LHID), which is randomly abstracted from the National Health Insurance Research Database. The health insurance system in Taiwan enrolls more than 99% of the population (i.e., more than 23,000,000 insurants per year). The LHID includes the characteristics of outpatients, patients seen in emergency departments, and inpatients. Among all insurants, the LHID randomly collected data on 13,139,306 insurants with 45,900,316 medical events from 1 January 2000 to 31 December 2013. The composition and characteristics of the individuals in the LHID were normally distributed.

The authors utilized the *International Classification of Disease, Ninth Revision, Clinical Modification* (ICD-9-CM) coding system to identify diagnoses and related procedures. Choledocholithiasis is defined as ICD-9-CM code 574. We enrolled all patients diagnosed with choledocholithiasis between 2000 and 2013 who also underwent any kind of invasive procedures (i.e., OC, LC, EC, or CEOC) during hospitalization. We excluded patients younger than 18 years of age and insurants with a history of choledocholithiasis before 1 January 2000.

The choledocholithiasis patients were divided into four groups based on the ICD-9-CM procedure and National Health Insurance Order codes. OC is defined as 51.41 and choledocholithotomy and T-tube drainage (75209B). LC is defined as 51.96 and laparoscopic choledocholithotomy (75218B). EC is defined as 51.88 and endoscopic sphincterotomy (EST) (56031B), endoscopic balloon sphincteroplasty (56032B), or endoscopic papillotomy with stone extraction (56033B). CEOC is defined as primary EC failure and necessity for conversion to OC, which combines both EC and OC during a single admission. The baseline characteristics were provided by the LHID. The study protocol was approved by the institutional review board of Tri-service General Hospital, Taiwan, Republic of China (approval no. TSGH-IRB No 2-105-05-082 and TSGH-IRB No. B-109-27).

We defined treatment-related complications as those that occurred within 30 days after treatment. Patients who were readmitted within 90 days after treatment were defined as readmission. Patients who received one of three treatments during readmission were defined as retained stones. Patients who died after treatment during the study period were defined as cases of mortality. All treatment-related complications were identified using ICD-9 codes (Table 1).

Statistical analyses were performed using IBM SPSS for Windows, version 20.0 (IBM Corp., Armonk, NY, USA). Chi-square and Fisher’s exact tests were used to compare categorical variables. Kaplan-Meier curve analysis and log-rank tests were used to demonstrate the cumulative risk for subsequent complications, readmission, re-treatment, and mortality. The hazard ratios (HRs) of subsequent complications and the other parameters of interest were calculated by multivariate Cox regression analysis. The relative risks (RRs) of hospital stay and medical costs were calculated by linear regression analysis. *p* values less than 0.05 were considered statistically significant.

## 3. Results

**Figure 1:**
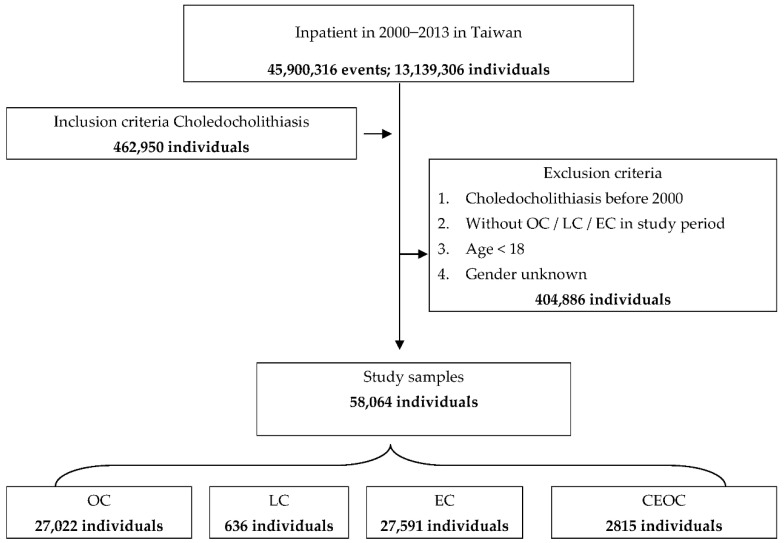
Flowchart of study sample selection from the National Health Insurance Research Database in Taiwan. Choledocholithiasis was defined as *International Classification of Disease, Ninth Revision, Clinical Modification* (ICD-9-CM) code 574. We enrolled all patients diagnosed with choledocholithiasis between 2000 and 2013 who received any kind of invasive procedure (i.e., OC, LC, EC, or CEOC) during hospitalization. We excluded patients younger than 18 years and insurants with a history of choledocholithiasis before 1 January 2000.

After applying the exclusion criteria, 404,886 patients were removed from the analysis; thus, only 58,064 patients were selected for subsequent analysis. The research flowchart is presented in Figure 1. The baseline patient characteristics are also shown (Table 2). Patients who underwent LC were older than those who underwent OC, EC, and CEOC (*p* < 0.001). The patients who underwent EC had more catastrophic illnesses and chronic obstructive pulmonary disease (COPD). The patients who underwent CEOC had more diabetes mellitus (DM), hypertension (HT), chronic kidney disease (CKD), and CHF (chronic heart failure).

Table 3 shows the endpoint characteristics of the study, including complications, readmission, retained stones and retreatment with OC or LC, retreatment with EC, mortality, hospital stay, and medical costs. The LC group had higher readmission, longer hospital stay, and higher medical cost than those of the other groups (*p* < 0.001). More patients with retained stones in the LC group received OC or LC as a secondary treatment, but patients with retained stones in the CEOC group received EC as secondary treatment (*p* < 0.001). More LC group patients had catastrophic illness compared to the other three groups (*p* < 0.001). There were significant differences in the incidence of comorbidities in the LC group, including DM, HT, CKD, CHF, and COPD.

Table 4 shows the rate of treatment-related events during the 14-year follow-up period. At the end of the follow-up period, there were 3070 treatment-related complications, 6986 readmissions, 650 OC or LC for retained stones, 1948 EC for retained stones, and 212 cases of mortality. The EC group had the highest complication, readmission, and mortality rates. The LC group received more OC or LC for retained stones, while the CEOC group received more EC for retained stones. Table 5 shows the results of Cox regression analysis of the risk factors associated with treatment-related complications within 30 days. After adjusting for insurance premium level, DM, and urbanization, the adjusted HR of EC was 1.259, higher than that of OC. Men were at a higher risk for complications than women. Patients with CKD had a higher risk of developing complications.

Table 6 shows the results of Cox regression analysis of the risk factors associated with treatment-related retained stones within 90 days and re-treatment with surgery (OC or LC) and endoscopy (EC). After adjusting for insurance premium level and sex by multivariate Cox regression, the adjusted HRs of readmission of LC, EC, and CEOC were higher than that of OC. The adjusted HRs of re-treatment with surgery of LC or EC were higher than that of OC. The adjusted HRs of re-treatment with endoscopy of EC and CEOC were higher than that of OC.

Table 7 shows the results of Cox regression analysis of the risk factors associated with mortality, hospital stay, and medical cost. After adjusting for insurance premium level, urbanization level, and hospital level of care by multivariate Cox regression, the adjusted HR of mortality of EC was higher than that of OC. The adjusted HRs of hospital stay in the LC and CEOC groups were higher than that of OC. Men, patients with catastrophic illness, and patients with DM and CKD were associated with a longer hospital stay. In addition, higher urbanization was associated with a longer hospital stay. The adjusted HR of medical costs for LC was higher than that for OC. Men, patients with catastrophic illness, and patients with DM and CKD were also associated with higher medical costs. Higher urbanization and care in a hospital center were also associated with higher medical costs.

## 4. Discussion

The ideal treatment for choledocholithiasis remains controversial [5]. Before the advent of laparoscopy and endoscopic methods, OC and common bile duct (CBD) exploration were the standard treatment for patients with choledocholithiasis. With the emergence of endoscopic retrograde cholangiopancreatography (ERCP) in the 1970s, EST has become the most common intervention for choledocholithiasis [10,11]. With increasing skills in laparoscopic surgery, laparoscopic CBD exploration can be technically demanding and may include extensive manipulation as well as laparoscopic suturing of the CBD. However, in the last decade, laparoscopic CBD exploration (LCBDE) has become the treatment of choice for choledocholithiasis in expert hands due to its advantages over the open and endoscopic methods [6,12]. This study investigated whether different choledocholithiasis treatments could independently result in different patient outcomes. In general, the baseline characteristics of each group were somewhat different. Most patients were older than 60 years of age, which was compatible with the ages reported in previous studies [13,14]. We also found that choledocholithiasis patients who underwent different treatments had different outcomes, including complications, readmission rates, retained stones with re-treatment by surgery or endoscopy, and mortality rates. These results are not consistent with previous studies that showed no significant difference in the mortality, morbidity, retained stones, and failure rates between different treatments [5,6,7,8,9].

Current evidence suggests that laparoscopic CBD stone clearance is as efficient as ERCP and EST, resulting in a reduced number of total procedures, shorter hospital stay, and similar mortality and morbidity rates [7,8,9]. However, most patients in our study underwent OC and EC and only 1.10% of patients underwent LC. There are several possible explanations for these findings. First, LC is technically demanding and was not well established in Taiwan in that period. Some surgeons are not convinced that LC and its surgical outcomes are as good as other treatments. Second, most choledocholithiasis patients are under the care of gastroenterologists who prefer EC except for cases of rare failure-related factors such as postsurgical gastrointestinal anatomic variations (Billroth II), duodenal diverticulum, embedded stones in the ampulla, intrahepatic bile duct stones, and CBD strictures. Third, patients with choledocholithiasis may hesitate to undergo surgical treatment.

This 14-year follow-up study observed different rates of complications, readmission, re-treatment for retained stones, and mortality, as well as hospital stay and medical costs among treatment groups (Table 3). Because the patients in our study who underwent LC were older and had more comorbidities, LC might have higher readmission, higher medical costs, and longer hospitalization than those of the other treatment groups. Previous study findings suggest that LC is safe and efficient. This method also provides single-stage management of cholelithiasis and choledocholithiasis with minimum morbidity and all the patient advantages of minimal access surgery [2,4,12]. Although LC is less invasive, patient selection, procedure duration, and postoperative care should be considered. To make LC better, the learning curve, education, and evolving laparoscopic techniques may also play important roles.

ERCP with EST has been available in most major medical centers worldwide for nearly 30 years [15,16] and is routinely used in conjunction with laparoscopic cholecystectomy, rather than OC for the treatment of choledocholithiasis. The overall success rate of ERCP in experienced hands is approximately 95%. However, the minimum number of ERCP procedures necessary for competency reported by Jowell et al. [17] and Vitale et al. [18] is between 102 and 185 procedures for a success rate of 85% to 90%. Table 2 showed that more patients with comorbidities underwent EC or CEOC because ERCP with EST was less invasive than surgical treatment. Most doctors would choose it as the first option. If EC failed, the patient would convert to the CEOC group. Therefore, as shown in Table 4, the EC group had higher rates of complication, readmission, and mortality. Assessment of complications revealed higher risks of complications in the EC group and patients with fewer comorbidities. Male and young patients also had higher risks of complications (Table 5). In addition, the EC group had a higher risk of mortality (Table 7). In clinical practice in Taiwan, more than 50% of choledocholithiasis patients undergo EC first, especially those with higher numbers of comorbidities and older patients who cannot tolerate surgical and anesthesia risks. From our results, EC is a somewhat risky procedure. More recently, a critical meta-analysis appraisal of the results of EST also showed morbidity rates of 5 to 11% and a mortality rate of less than 1% [5], mostly due to acute pancreatitis, duodenal perforation, sepsis, and bleeding. However, our retrospective study has some limitations and bias, further studies to prove this point of view is mandatory.

Several studies have reported on the efficacy, safety, and efficiency of CBD stone removal by ERCP and LCBDE [2,5]. In our study, the total mortality of the choledocholithiasis treatments was 0.36%, comparable to that of a previous study [5], and male sex, old age, and patients with a catastrophic illness also had higher mortality rates (Table 7). The morbidity rates for different choledocholithiasis treatments were 4.3–16% [5] and they were similar in our OC, LC, EC, and CEOC groups, respectively (Table 3). Although data regarding quality of life and procedure duration were not included in our Taiwan LHID, our results suggest the quality of different treatments for choledocholithiasis in Taiwan is acceptable.

Retained or recurrent stones are also an important issue in the treatment of choledocholithiasis. A previous study [19] defined retained stones as stones detected within one year after the index treatment and recurrent stones as stones found one year after the index treatment. Whether choledocholithiasis is detected after the index treatment is considered retained or recurrent stones remain uncertain. Some studies suggested retained stones were diagnosed by completion cholangiography and choledochoscopy during the index operation, and postoperative T-tube cholangiography or occasionally postoperative ERCP after surgery. The clearance rate of LCBDE in previous studies was 100%, likely due to the meticulous attention to detail paid by the surgeon in checking for residual stones in the CBD using intraoperative cholangiography or choledochoscopy [20,21]. In a meta-analysis of seven trials including 609 participants, those who underwent open surgery had significantly fewer retained stones compared with those who underwent ERCP [5]. It is important to remember that these comparative trials are from the early days of endoscopy (1987 to 1998) and might have been influenced by the early experience of the endoscopist as well as the limited technological support. Another meta-analysis compared retained stones of laparoscopic cholecystectomy and LCBDE with those of pre-operative ERCP and laparoscopic cholecystectomy for cholelithiasis and choledocholithiasis [5]. There was no significant difference in the retained stones between the two groups. In our data, retained stones were less than 10% in each group. The Taiwan LHID does not contain data on the methods and tools used to evaluate the CBD clearance of the index treatment; this may be related to the higher rate of retained stones. From our data, previous LC patients more commonly underwent LC or OC for retained stones, while previous CEOC patients more often underwent ES for retained stones. Although patients can discuss the treatment with their doctors, the treatment usually depends on the doctors. When a surgeon chose LC as the first treatment, they had an increased likelihood of choosing surgery such as LC or OC for re-treatment. Similarly, gastroenterologists also tried ES for re-treatment, converting to CEOC if the treatment failed. In this way, there might be a little risky not to resolve the retained stones again if patients receive the same treatment.

A previous study reported an ERCP success rate of 88.1%. The main reason for unsuccessful clearance was impacted stones in 13.1% of patients. Difficulty with cannulation and impacted stones were the common causes of treatment failure [22]. The independent determinants for failed laparoscopic CBD stone removal included stone size ≥7 mm; a transductal approach; and difficult cystohepatic triangle anatomy due to adhesions, scarring, and fibrosis [19]. Hong et al. [6] reported the results of a trial assessing laparoscopic cholecystectomy + LCBDE versus laparoscopic cholecystectomy + intra-operative ERCP. There was no significant difference in procedure failure rates between the two intervention groups. If patients failed their initial treatment and received another treatment during the same admission, such as the CEOC group in our study, which we considered to be treatment failures. From our data, the incidence of CEOC was 4.8%. Most patients failed the EC treatment and underwent OC thereafter. Although OC was considered the standard salvage treatment, some patients still experienced retained stones (6.8%); thus, LC might be an alternative choice for treatment failure.

The strengths of our study include its use of national data with a large sample size and the presentation of the incidence trends in the most recent decade. However, it also had some limitations. First, the database cannot show an association among timing, indication, and choledocholithiasis treatments. We cannot predict which individuals with different severities of choledocholithiasis will benefit from OC, LC, or EC. Consequently, some individuals experienced complications, readmission, or treatment failure. Further prospective studies are necessary to better understand the indications for different choledocholithiasis treatments in Taiwan. Second, data regarding patient quality of life after different treatments were lacking in this study. The LHID does not contain detailed information about how patients felt following treatment. Further prospective randomized control studies with well-designed questionnaires should focus on determining whether the quality of life after treatment plays a role in the selection, quality, and success of different treatments. Third, our database did not contain data on the recurrence rate after each treatment. Recurrence also plays an important role in treatment success; however, there is currently no clear definition of recurrence. A longer follow-up period may be necessary to clarify the long-term outcomes and quality of each treatment for choledocholithiasis in Taiwan. Fourth, the LHID files did not provide information regarding family history, physical activity, and dietary habits, all of which might be risk factors for choledocholithiasis. Therefore, further prospective studies are required to better understand the relationships between these factors and choledocholithiasis in Taiwan.

## Figures and Tables

**Table 1:** ICD-9 codes of treatment-related complications.

ICD-9-CM List	
Comorbidity	ICD-9-CM
Diabetes mellitus (DM)	250
Hypertension (HT)	401–405
Depression	296.2–296.3, 296.82, 300.4, 311
Chronic kidney disease (CKD)	585
Congestive heart failure (CHF)	428
Chronic obstructive pulmonary disease (COPD)	490–496
Hyperlipidemia	272
Complication	ICD-9-CM
Intestinal infections due to other organisms	008.0–008.8
Ill-defined intestinal infections	009.0–009.1
Bacterial infection in conditions classified elsewhere and of unspecified site	041
Acute respiratory failure	518.81–518.84
Perforation of the esophagus	530.4
Gastroesophageal laceration-hemorrhage syndrome	530.7
Gastric ulcer	531
Duodenal ulcer	532
Peptic ulcer	533
Gastrojejunal ulcer	534
Gastritis and duodenitis	535
Disorders of the function of the stomach	536
Other hernia of the abdominal cavity	551.1–553.9
Intestinal obstruction without mention of hernia	560
Other disorders of the peritoneum	568
Other disorders of the biliary tract	576–577
Gastrointestinal hemorrhage	578
Acute renal failure	584.5–584.9
Shock without mention of trauma	785.50–785.51
Other shock without mention of trauma	785.59
Injury to intra-abdominal organs	863.0–868.19
Late effect of complications of surgical and medical care	909.3

**Table 2:** Baseline study characteristics.

Surgery	Total		OC		LC		EC		CEOC		*p*
Variables	*n*	%	*n*	%	*n*	%	*n*	%	*n*	%	
Total	58,064		27,022	46.54	636	1.10	27,591	47.52	2815	4.85	
Gender											0.072
Male	30,225	52.05	13,926	51.54	346	54.40	14,496	52.54	1457	51.76	
Female	27,839	47.95	13,096	48.46	290	45.60	13,095	47.46	1358	48.24	
Age (years)	64.27 ± 14.69		64.76 ± 14.02		65.66 ± 13.48		63.71 ± 15.36		64.60 ± 14.33		<0.001
Catastrophic illness	3058	5.27	1492	5.52	40	6.29	1387	5.03	139	4.94	0.035
DM	8609	14.83	3726	13.79	81	12.74	4367	15.83	435	15.45	<0.001
HT	10,740	18.50	4420	16.36	101	15.88	5747	20.83	472	16.77	<0.001
CKD	536	0.92	210	0.78	6	0.94	306	1.11	14	0.50	<0.001
CHF	661	1.14	273	1.01	7	1.10	340	1.23	41	1.46	0.034
COPD	1611	2.77	787	2.91	26	4.09	715	2.59	83	2.95	0.021

(Categorical variables: Chi-square/Fisher exact tests; continuous variables: One-way analysis of variables (ANOVA) with Scheffe post hoc tests). open choledocholithotomy (OC), laparoscopic choledocholithotomy (LC), endoscopic choledocholithotomy (EC), combined endoscopic and open choledcholithotomy (CEOC).

**Table 3:** Characteristics of study in the endpoint.

Surgery	Total		OC		LC		EC		CEOC		*p*
Variables	*n*	%	*n*	%	*n*	%	*n*	%	*n*	%	
Total	58,064		27,022	46.54	636	1.10	27,591	47.52	2815	4.85	
Complications within 30 days	3070	5.29	1448	5.36	38	5.97	1464	5.31	120	4.26	0.069
Readmission within 90 days	6986	12.03	2549	9.43	119	18.71	3999	14.49	319	11.33	<0.001
Retreatment with OC or LC within 90 days	650	1.12	276	1.02	36	5.66	311	1.13	27	0.96	<0.001
Retreatment with EC within 90 days	1948	3.35	881	3.26	17	2.67	885	3.21	165	5.86	<0.001
Mortality	212	0.37	110	0.41	3	0.47	88	0.32	11	0.39	0.295
Hospital stay (days)	59.01 ± 106.34		66.86 ± 113.10		73.64 ± 108.05		48.15 ± 91.94		71.04 ± 140.82		<0.001
Medical cost (NT$)	359,399.14 ± 460,707.83		406,813.02 ± 503,114.00		414,664.61 ± 536,972.69		299,933.29 ± 292,122.45		396,330.71 ± 487,416.80		<0.001
Gender											0.072
Male	30,225	52.05	13,926	51.54	346	54.40	14,496	52.54	1457	51.76	
Female	27,839	47.95	13,096	48.46	290	45.60	13,095	47.46	1358	48.24	
Age (years)	68.18 ± 14.45		69.19 ± 13.78		69.31 ± 13.78		67.01 ± 15.14		68.05 ± 14.13		<0.001
Catastrophic illness	6262	10.78	3301	12.22	96	15.09	2575	9.33	290	10.30	<0.001
DM	10,388	17.89	5246	19.41	138	21.70	4541	16.46	463	16.45	<0.001
HT	15,783	33.10	7853	36.06	217	43.57	7031	30.50	682	29.00	<0.001
CKD	1640	2.82	832	3.08	27	4.25	725	2.63	56	1.99	<0.001
CHF	3396	5.85	1854	6.86	67	10.53	1305	4.73	170	6.04	<0.001
COPD	5357	9.23	3031	11.22	89	13.99	1985	7.19	252	8.95	<0.001

(Categorical variables: Chi-square/Fisher exact tests; continuous variables: One-way analysis of variables (ANOVA) with Scheffe post hoc tests). open choledocholithotomy (OC), laparoscopic choledocholithotomy (LC), endoscopic choledocholithotomy (EC), combined endoscopic and open choledcholithotomy (CEOC).

**Table 4:** Rate of events.

Events	Surgery	Event	PDs	Rate (per 10^5^ PDs)
Complication in 30-day tracking	OC	1448	386,306.53	374.83
	LC	38	11,157.62	340.57
	EC	1464	298,594.78	490.30
	CEOC	120	34,352.76	349.32
Readmission in 90-day tracking	OC	2549	17,697,264.77	14.40
	LC	119	476,691.95	24.96
	EC	3999	9,164,265.52	43.64
	CEOC	319	1,294,065.34	24.65
Retreatment with OC or LC in 90-day tracking	OC	276	19,656,255.02	1.40
	LC	36	545,878.95	6.59
	EC	311	10,400,418.59	2.99
	CEOC	27	1,466,331.67	1.84
Retreatment with EC in 90-day tracking	OC	881	19,086,052.40	4.62
	LC	17	561,947.45	3.03
	EC	885	10,110,443.22	8.75
	CEOC	165	1,382,956.77	11.93
		Event	PYs	Rate (per 10^5^ PYs)
Mortality	OC	110	54,273.14	202.68
	LC	3	1576.84	190.25
	EC	88	28,830.59	305.23
	CEOC	11	4037.42	272.45

PDs = Person-days, PYs = Person-years. open choledocholithotomy (OC), laparoscopic choledocholithotomy (LC), endoscopic choledocholithotomy (EC), combined endoscopic and open choledcholithotomy (CEOC).

**Table 5:** Factors of complication in 30-day tracking by using Cox regression.

Variables	Adjusted HR	95% CI	95% CI	*p*
Treatment				
OC	Reference			
LC	0.921	0.668	1.272	0.618
EC	1.259	1.170	1.355	<0.001
CEOC	0.889	0.737	1.071	0.215
Gender				
Female	Reference			
Male	1.195	1.112	1.285	<0.001
Age (years)	0.982	0.979	0.985	<0.001
Catastrophic illness	0.683	0.620	0.753	<0.001
DM	0.960	0.884	1.044	0.340
HT	0.859	0.792	0.931	<0.001
CKD	1.482	1.273	1.724	<0.001
CHF	0.801	0.698	0.919	0.002
COPD	0.805	0.721	0.900	<0.001

Adjusted HR (hazard ratio): Adjusted variables listed in the table, CI = confidence interval. open choledocholithotomy (OC), laparoscopic choledocholithotomy (LC), endoscopic choledocholithotomy (EC), combined endoscopic and open choledcholithotomy (CEOC).

**Table 6:** Factors of readmission and retreatment with OC or LC/EC in 90-day tracking by using Cox regression.

Events	Readmission				Retreatment with OC or LC				Retreatment with EC			
Variables	Adjusted HR	95% CI	95% CI	*p*	Adjusted HR	95% CI	95% CI	*p*	Adjusted HR	95% CI	95% CI	*p*
Treatment												
OC	Reference				Reference				Reference			
LC	1.624	1.351	1.952	<0.001	4.237	2.990	6.004	<0.001	0.622	0.385	1.005	0.053
EC	1.871	1.780	1.968	<0.001	1.415	1.202	1.666	<0.001	1.285	1.170	1.412	<0.001
CEOC	1.285	1.143	1.444	<0.001	1.018	0.685	1.513	0.929	1.891	1.600	2.235	<0.001

Adjusted HR (hazard ratio): Adjusted variables listed in the table, CI = confidence interval. open choledocholithotomy (OC), laparoscopic choledocholithotomy (LC), endoscopic choledocholithotomy (EC), combined endoscopic and open choledcholithotomy (CEOC).

**Table 7:** Factors of mortality/lengths of days/medical cost by using Cox regression/linear regression.

Events	Mortality				Log (Length of Days)				Log (Medical Cost)			
Variables	Adjusted HR	95% CI	95% CI	*p*	Adjusted RR	95% CI	95% CI	*p*	Adjusted RR	95% CI	95% CI	*p*
Treatment												
OC	Reference				Reference				Reference			
LC	0.979	0.310	3.095	0.971	1.237	1.157	1.321	<0.001	1.306	1.224	1.393	<0.001
EC	1.603	1.208	2.132	0.001	0.955	0.850	1.074	0.443	0.906	0.824	1.393	0.145
CEOC	1.388	0.745	2.587	0.302	1.124	1.089	1.160	<0.001	0.997	0.831	1.054	0.273
Gender												
Female	Reference				Reference				Reference			
Male	1.561	1.157	2.106	0.002	1.132	1.099	1.166	<0.001	1.133	1.100	1.167	<0.001
Age (years)	1.030	1.016	1.044	<0.001	0.999	0.988	1.001	0.112	0.999	0.998	1.001	0.128
Catastrophic illness	5.964	4.476	7.946	<0.001	1.269	1.228	1.331	<0.001	1.278	1.235	1.319	<0.001
DM	1.048	0.779	1.410	0.757	1.075	1.042	1.110	<0.001	1.059	1.026	1.093	<0.001
HT	0.626	0.467	0.840	0.002	0.800	0.775	0.826	<0.001	0.844	0.817	0.871	<0.001
CKD	1.428	0.941	2.167	0.094	1.137	1.074	1.204	<0.001	1.168	1.103	1.236	<0.001
CHF	1.085	0.742	1.587	0.674	0.968	0.927	1.011	0.148	0.965	0.923	1.008	0.107
COPD	0.928	0.667	1.293	0.660	0.973	0.937	1.010	0.157	0.939	0.904	0.975	0.001

Adjusted HR (hazard ratio): Adjusted variables listed in the table, CI = confidence interval, Adjusted RR (relative risk): Adjusted variables listed in the table. open choledocholithotomy (OC), laparoscopic choledocholithotomy (LC), endoscopic choledocholithotomy (EC), combined endoscopic and open choledcholithotomy (CEOC).

## Data Availability

All data are available in the text of the manuscript. Further anonymized data can be made available to qualified investigators upon reasonable request.
